# Guilt in Bereavement: The Role of Self-Blame and Regret in Coping with Loss

**DOI:** 10.1371/journal.pone.0096606

**Published:** 2014-05-12

**Authors:** Margaret Stroebe, Wolfgang Stroebe, Rens van de Schoot, Henk Schut, Georgios Abakoumkin, Jie Li

**Affiliations:** 1 Department of Psychology, Utrecht University, Utrecht, The Netherlands; 2 Department of Psychology, University of Groningen, Groningen, The Netherlands; 3 Department of Methods and Statistics, Utrecht University, Utrecht, The Netherlands; 4 Optentia Research Program, Faculty of Humanities, North-West University, Mahikeng, South Africa; 5 Department of Early Childhood Education, University of Thessaly, Volos, Greece; 6 Department of Psychology, Renmin University of China, Beijing, China; University of Utah, United States of America

## Abstract

Despite the apparent centrality of guilt in complicating reactions following bereavement, scientific investigation has been limited. Establishing the impact of specific components associated with guilt could enhance understanding. The aim of this study was to examine the relationships between two guilt-related manifestations, namely self-blame and regret, with grief and depression. A longitudinal investigation was conducted 4–7 months, 14 months and 2 years post-loss. Participants were bereaved spouses (30 widows; 30 widowers); their mean age was 53.05 years. Results showed that self-blame was associated with grief at the initial time-point and with its decline over time. Such associations were not found for depression. Initial levels of regret were neither associated with initial levels of grief and depression, nor were they related to the decline over time in either outcome variable. These results demonstrate the importance of examining guilt-related manifestations independently, over time, and with respect to both generic and grief-specific outcome variables. A main conclusion is that self-blame (but not regret) is a powerful determinant of grief-specific difficulties following the loss of a loved one. Implications for intervention are considered.

## Introduction

People often wish that they could have done things differently following the death of a loved one; this can make them feel guilty. For example, bereaved persons may think that they should have done more to prevent the death or to have lived up to their own expectations in their prior relationships with the deceased (e.g.,[1] [2]). Guilt is typically listed not only among reactions to the loss of a loved one [3] it is also an integral part of depression. Guilt in the bereavement context has been defined as “a remorseful emotional reaction in bereavement, with recognition of having failed to live up to one's own inner standards and expectations in relationship to the deceased and/or the death” [4](p. 166). Although there is some evidence that it has a detrimental impact on adjustment to bereavement, empirical investigation has been limited and results have so far been inconclusive. For example, although identified as associated with grief, its precise role in the development of severe complications in bereavement remains unclear [4]. Yet, establishing the relationship between guilt and complications/symptomatology would seem critical. For example, in the bereavement field, one of the foci in psychotherapeutic intervention programs for persons enduring complications in their grieving has involved altering persisting negative attributions, including those associated with guilt, self-accusations and remorse (e.g.,[1]). This would point to an assumed causal role of such reactions in grief complications.

Understanding the role of guilt in adjustment to bereavement is complicated by the fact that guilt incorporates a variety of cognitive and emotional components [4], suggesting the need for finer-grained examination. The present study focuses on two components of guilt, namely, self-blame and regret. There are good reasons to select these two: They are the most-frequently identified forms of guilt in the bereavement literature (reviewed below), yet systematic comparisons of their impact have so far been lacking. Given the specific characteristics of self-blame and regret, one might assume that they have different associations with psychological well-being among bereaved persons: Self-blame and regret are close, yet distinct phenomena, ones which may play different roles in the adaptation process after loss.

To elaborate: Self-blame in the bereavement literature usually refers to making self-attributions about the cause of the death (e.g.[5],[6]), and a sense of culpability due to failure to live up to standards of the deceased or one's self [7],[8]. Regret has been identified in the general literature as involving painful thoughts and feelings about past actions and how one could have achieved a better outcome [9], and in the bereavement specific literature, as feelings associated with unfinished business with the deceased in general [10], or the perception that one could have done things differently [11]. Accordingly, we define regret in the context of bereavement, as a negative emotion accompanied by the belief that one could have done something differently to bring about a more desirable outcome with respect to the relationship with the deceased and/or the death-related events.

As evident from the above definitions, while self-blame stresses responsibility for the death, and implies accusation of oneself, regret in bereavement focuses more on possible better outcomes, without impaired sense of self. Negative cognition focused on oneself, as in self-blame, has been suggested to play a more detrimental role in psychological well-being [12],[13],[14] and adjustment in grief [15] than negative perception of one's behaviour or the event per se, as in regret. A major purpose of the current longitudinal investigation is, then, to compare the role of self-blame with that of regret in the process of coming to terms with the loss of a loved one. We examine their role not only as determinants but also their course over time, across the months of acute grief and grieving.

Next we examine the extent to which relationships between these two phenomena and health outcomes have been established so far in the bereavement literature.

### The role of self-blame in bereavement

Some studies have reported a negative influence of self-blame on grieving people's well-being. Associations between these variables have been found in some cross-sectional studies. Hazzard et al. [16] conducted a study among bereaved parents and concluded that higher self-blame for the death was associated with more intense grief reactions, as measured using the Grief Experiences Inventory [17]. However, this study included only one item to measure participants' self-blame for the death, raising concerns about the reliability and validity of their assessments of self-blame and resulting conclusions. Using a somewhat better measure, Garnefski and Kraaij [18] adopted the self-blame subscale from an emotion regulation scale to investigate the concurrent relationship between self-blame in bereavement and depressive symptoms. These investigators also found positive correlations between these variables.

Relating self-blame to a somewhat different measure of the course of grief, in two earlier studies by Weinberg [6],[19], participants were asked to indicate their level of “recovery” from their loss: to what extent they thought they had “got over” the death. Participants who blamed themselves more for the death also reported poorer recovery. Again, these studies also relied on single item measures at a single time-point.

More recently, adopting a measure with better psychometric properties, the self-blame subscale of the Grief Cognition Questionnaire, Boelen and colleagues [15],[20] found that higher self-blame was correlated with higher psychological distress (using the depression subscale of the SCL and anxiety subscales of the SCL-90), as well as more severe grief reactions (on an established grief scale, see [21]). However, these investigations were also cross-sectional, so no statements could be made about the impact of self-blame on grief (or vice versa).

The strongest evidence comes from longitudinal investigations, given that causal connections can be more firmly established than in cross-sectional ones. The available longitudinal studies have focused on whether self-blame during the early stages of bereavement predicts later adjustment. In a classic early study by Horowitz et al [22], attributions of blame for the death were investigated. Bereaved adult children with severe grief who had sought (and were receiving) treatment were compared with a bereaved non-patient, so-called “field”, control group. Participants' self-blame and psychological distress were evaluated over time until just over a year after loss. Psychological distress was assessed using a battery of symptom measures, including clinicians' ratings and scales such as the SCL-90 and Impact of Event Scale. Those who attributed more responsibility for the death to themselves, showed a slower decline in psychological symptoms, the effect being stronger for patients than field participants [22]. This study investigated self-blame as a predictor, not its course over time. Thus, only self-blame assessed at the first point in time was used to predict the decline of psychological distress. Furthermore, only general mental health, but not grief reactions, was examined. Finally, a point which applies to studies using the total SCL, is that this symptoms list contains two (or one, in short versions) items on guilt: blaming oneself for a variety of things and feeling guilty. So, unless excluded (to our knowledge only Boelen et al. [15] did so), there is conceptual overlap between this and guilt measures, which would increase the likelihood of a positive relationship being found between these variables.

Two more recent studies by Field and colleagues, using a very different methodology, have provided further evidence regarding the predictive value of self-blame on adjustment to bereavement [8],[7]. Field et al. examined the content of bereaved spouses' monologues directed toward the deceased, 3–7 months after the death [8]. A higher degree of self-blame as coded from the spouses' narratives predicted higher grief symptoms (but not depression) at 14 months. In the Field and Bonanno follow-up investigation, it was found that self-blame 6 months after bereavement predicted grief symptoms as long as 5 years after the loss [7]. These studies were stringent in controlling for symptom levels at the first point of measurement. However, their index of self-blame was based on judges' assessments and not psychometrically-tested rating scales.

Not only are there methodological limitations in studies claiming a relationship between bereaved peoples' self-blame and general psychological distress, but some researchers have failed to find a negative influence between these two variables. Downey, Silver, and Wortman traced a group of bereaved parents at one, three and 18 months after the death of their child [23]. The concurrent correlation between self-blame and psychological distress (as measured on a shortened version of the SCL-90) was significant, while the longitudinal association was not. This included the entire depression subscale, which again contains one item on guilt (which was not deleted). Downey and colleagues concluded that there was no causal relationship between self-blame and maladjustment in bereavement. It is possible that self-blame was assessed too early in their study to have predictive validity, since it is common for people to experience high distress and negative cognitions soon after the death, which attenuate with time [24]. In fact, most of the studies assessed self-blame after at least 6 months. It is also noteworthy that the measure was not grief-specific but one “indexing generalized distress” [23](p. 929). By contrast, another more recent study compared people diagnosed with complicated grief with a group of healthy controls [25]. Here too, though, when self-blame was the focus, no significant difference between these groups was found. The study was cross-sectional. Taken together, the negative results reported in these investigations do not provide strong evidence against the hypothesis that self-blame is associated with higher general symptomatology or grief.

### The role of regret in bereavement

Compared with the empirical studies on self-blame, there have been even fewer studies focusing on regret in bereavement. Again, longitudinal designs provide more conclusive evidence on the causal relationships between these variables than cross-sectional ones. One longitudinal study by Torges, Stewart, and Nolen-Hoeksema asked participants “Are there things you wish you had done differently?”, and found that reported regret was positively associated with depression [11]. Furthermore, the interaction between regret and time significantly predicted decrease in depressive symptoms. However, another recent study by Holland et al. [10] suggested that it is important to take the trajectory of regret over time into account in association with grief symptoms. These investigators measured grief reactions and frequency of regret felt by bereaved participants at four, 18 and 48 months after loss. They divided their participants into three groups, stable low regret, stable high regret, and worsening high regret. The worsening high regret group showed higher levels of grief symptoms at all three points in time and at 48 months, even higher levels than the stable high regret group. None of the regret trajectories was related to differences in depressive symptomatology.

Two cross-sectional studies [26],[27] have reported positive association between regret and psychological distress (including depression), and grief. However, both of these investigations combined items of regret and self-blame into one measure, thus giving little information on the unique role of regret. Moreover, only Japanese participants were included in these studies, which - while usefully extending investigation to another culture - raises the question about comparability of their findings with those of other studies, which have typically been conducted in western countries.

### Conclusions

In summary, the results on the role of either self-blame or regret in bereavement have been quite discrepant, making it difficult to draw overall conclusions from the available literature. With respect to self-blame: Some investigations have suggested that it impacts on grief, with perhaps the strongest evidence coming from the Field studies[8],[9]. A relationship between self-blame and more general symptoms (depression in this study) was not found by these investigators, but others, such as Horowitz et al. [17], have found associations between such variables. Turning to regret: There are some indications of an association of regret with depression and with grief reactions, but firm conclusions are difficult to draw from the sparse literature specifically on regret.

In general, longitudinal investigation on the impact of either self-blame or regret on adjustment to bereavement has been scarce. The few available investigations over time have measurement shortcomings (e.g. single item measure and mixed items of self-blame and regret). Another shortcoming (linked to the general lack of longitudinal studies) is the paucity of information on the course of the self-blame and regret manifestations over time, for example, regarding whether they actually decline. Finally, some investigations have focused on grief-specific, others on more generic (e.g., depression) outcome variables with possible overlap of guilt in the latter studies. Although sadness and depression are important symptoms of grieving, they are neither the only nor necessarily the most important ones. There is some evidence that depression and grief are influenced by different aspects of the marital relationship. For example, Stroebe, Abakoumkin and Stroebe found that marital quality affected only yearning for the loved one who died, but not depression, whereas experiencing support from family and friends reduced depression but did not ameliorate yearning[28]. Therefore, there seem to be good reasons to include both types of outcome variables within one investigation.

### The present study

Collection of data at multiple time points will permit examination of trajectories of self-blame and regret, enabling comparisons with the course of outcome variables. Tracing the pattern of change across time will provide more reliable information on the relationship between self-blame and regret with adjustment in bereavement. Furthermore, as indicated above, no study so far has assessed self-blame and regret separately (but in the same study) to explore their possible differential impact on adjustment to bereavement. Finally, we included both grief and depression as outcome measures. There are good reasons to include a bereavement-specific as well as a generic indicator of adjustment over time. While associations have been found between outcomes, different patterns of response have also been documented[29]. Our study was therefore designed to investigate both the effect of initial level of self-blame/regret and the influence of their trajectories across time on grief and depression.

Thus, the general aim of the current investigation is to contribute to the body of literature on the role of two major manifestations of guilt by improving on previous studies in a number of respects: It draws data from a carefully-controlled longitudinal study of widowed persons over the course of the first two years of their bereavement. It also includes quantitative measures of self-blame and regret, and it investigates the role and pattern of these manifestations separately and in relation to adjustment to loss over time. Furthermore, the study uses a statistical approach novel to bereavement research (latent growth modeling (LGM) e.g., [30]) to investigate individual symptom trajectories and predictors of outcome of bereavement. Finally, as noted above, in accordance with a few other studies [6],[10],[12],[17],[18], but in contrast to the majority that have focused on either depression or grief, our study includes both these variables. We consider it important to examine the impact of self-blame and regret on the course of bereavement-specific grief as well as more generic depression reactions. However, on the basis of the discussed literature it is hard to formulate strongly stated hypotheses. With regard to general distress or depression, we have no specific expectations about the relationship of depression with self-blame and regret (if at all, similar relationships would be expected). In contrast, it seems that self-blame is likely to have more severe repercussions than regret on grief, as a bereavement specific outcome. Therefore, we would expect self-blame to be associated with grief whereas this would not necessarily be the case for regret.

## Method

### Participants and procedure

Data for this investigation were drawn from the Tübingen Longitudinal Study of Bereavement, an in-depth study of conjugal bereavement conducted in Southern Germany (e.g., [31],[32],[33]). Thirty widows and thirty widowers (mean age 53.05 years, *SD*  = 6.81), who lost their spouse in the previous four to seven months participated in the study. Widowed individuals were approached through contact data that were provided by local registrars' offices. They were first sent a letter asking for their participation followed by a phone call. The acceptance rate that resulted (28%) was typical in the context of bereavement research [34].

The study included three time points of data collection: (a) 4–7 months after participants lost their spouse; (b) about 14 months post-loss; and (c) about 2 years post-loss. At the first and second time points data were collected at participants' homes, while at the third time point data collection was organized by telephone. At all time points participants had to respond to questionnaires (handed to them at T1 & T2; sent to them at T3) comprised of self-report measures (including the scales reported here) and semi-structured interviews. These questionnaires were completed (alone) after the interviews and returned by mail (in prepaid envelopes). With 82% participating at all three time points, drop-out rates were low. There was no significant difference in health between those who continued participation in all three sessions and those who dropped out [33]. It appeared there was also no significant effect between cases with and without missing data on the variables measured at wave 1 (Wilks' Lambda  = 0.864, *F*(4, 54)  = 2.12, *p* = .09), nor was there a significant gender difference, χ^2^ (1, *N* = 60)  = 1.667, *p* = .197.

### Ethical statement

The study was conducted in accordance with the ethical principles of the German Research Association (Deutsche Forschungsgemeinschaft) and the University of Tübingen, Germany, where the principle investigators were located at that time. No institutional review board existed in this country at the time when the data were collected (1983–1985). Also in accordance with the existing regulations, oral consent was obtained and documented securely and separately from the questionnaire and interview material, to ensure anonymity. Participants, who were drawn from the normal (not a clinical) population, were ensured of both anonymity and confidentiality. No pressure was put on persons to participate. They were carefully informed in writing and by telephone about the goal and scope of the research; it was made clear that this investigation was not an intervention/treatment study. They were also informed that they could discontinue their participation at any time, without any consequences.

### Measures

#### Identification of Guilt, Self-Blame and Regret Items

The first step toward development of the self-blame and regret scales was to identify those items on the Tübingen Bereavement Symptoms Questionnaire (TBSQ, unpublished data) which measured cognitions/emotions relating to guilt. The TBSQ was originally constructed as a comprehensive inventory of grief symptoms for the Tübingen Longitudinal Study of Bereavement mentioned above. For the current investigation, all 198 scale items on this scale were independently evaluated by three bereavement researchers. Items were selected on which there was agreement between the three judges; 10 items were identified that fell within the general category of guilt-related reactions. The 10 selected items relating to guilt were further assessed, this time by five bereavement researchers, who judged each item as a measure of self-blame/guilt or regret, in line with the definitions of these constructs. However, the final assignment of items to the two categories was made on the basis of factor analysis.

The self-blame and regret measures were correlated *r*(60)  = .48, *p*<.001, (Time 1). To examine whether these scales should be treated as different dimensions or not, all the 10 scale items were submitted to a factor analysis (principal components, varimax rotation). A clear two factor solution emerged; these factors accounted for 56.38% of the variance. Except for some minor cross-loadings, the items loaded on the two factors reflecting two different dimensions (see Table 1). Specifically, items (f) to (j) loaded on the first factor (Regret; eigenvalue 4.22, 42.21% of variance) and items (a) to (e) loaded on the second factor (Self-blame; eigenvalue 1.42, 14.17% of variance). Therefore, the self-blame and regret measures could be assumed to be conceptually distinct.

**Table 1:** Factor Loadings for the Self-Blame and Regret Items.

	Factor Loading	
Scale and Item	F1	F2
Self-Blame		
(a) I often wish I could have died instead of him.		.70
(b) Sometimes I have the feeling that I share responsibility for his death.		.72
(c) I've nothing to blame myself for, because I cared for him the whole time. (R)		.70
(d) I have guilt feelings because I'm sometimes able to enjoy life again.		.47
(e) I think I did everything for him that I could do. (R)	.49	.61
Regret		
(f) I often wish I could turn the clock back and do things differently.	.79	
(g) If I could be with him one more time, I'd do a lot differently.	.80	
(h) I really regret not having done more for him when he was alive.	.74	
(i) I really regret not always behaving well toward him.	.87	
(j) I have guilt feelings when I think of some of the things that I did while he was still alive.	.58	.44

*Note*. Only factor loadings with an absolute value greater than .30 are displayed. “(R)” denotes items that were reverse-coded for the analyses.

#### Self-blame

Self-blame was comprised of five items from the Tübingen Bereavement Symptoms Questionnaire (TBSQ), namely items (a) to (e) as shown on Table 1, which had a true-false format. The resulting scale had a satisfactory internal consistency (Time 1: α = .68).

#### Regret

Five items from the TBSQ were used to assess regret, i.e. items (f) to (j) as shown on Table 1; these items had a true-false format. The resulting scale also had a good internal consistency (Time 1: α = .84).

#### Grief

The Tübingen Grief Scale (TGS) was used (see [31]). This scale was included in the TBSQ. It is comprised of 13 items (e.g., “Sometimes I long for him so much that I can't think of anything else.”), with a true-false response format. It was designed for use in Germany. Selection of the items was guided by the work of Prigerson and colleagues being similar to those on their Inventory of Complicated Grief and somewhat paralleled their proposed criteria for a diagnostic category of Prolonged Grief Disorder [35]. The TGS had a good internal consistency (Time 1: α = .80).

#### Depression

The German version of the well-established Beck Depression Inventory (BDI [36]) was used. Originally, the BDI was comprised of 21 items assessing major depressive symptoms. The item “lack of sexual interest” was not included in the administered version, because, according to a pre-test, this item might have been perceived as offensive by the recently bereaved participants in our sample. In addition, the item “guilt feelings” was removed from the scale for the present analyses, due to conceptual overlap with the self-blame and regret scales (Time 1: α = .86). Depression and grief were significantly correlated (Time 1: *r*(59)  = .72, *p*<.001).

### Analytic strategy

To answer our research questions we used a novel statistical approach, latent growth modeling (LGM; e.g. [30]) to estimate individual trajectories over time. With this approach each individual can have his/her own starting point (i.e. a random intercept model) and development over time (i.e., random slope). In a next step predictors for the intercept and slope can be added to explain individual variation (i.e., explained variance). Use of this technique is quite new to bereavement research, it has not been adopted to investigate our specific research questions, while it incorporates features that, in our view, improve their scientific investigation. More specifically, it allows us to relate individual levels of self-blame and regret at the beginning of the study to individual trajectories of grief and depression over the three measurement points of our study. This enables examination of the association between individual differences in self-blame and regret (bereaved individuals begin with different starting points) to the individual course of grief and depression. If LGM is combined with Bayesian statistics, the method can be used for fairly small data sets [37], [38]; we will come back to this issue in the discussion section.

We used the software M*plus* v7.11 to run the LGM models [39]. For the Bayesian estimator we relied on the default settings as described in Muthén and Asparouhov [39], but we decreased the Gelman-Rubin [40] criterion for assessing convergence (.01 instead of .05), we increased the number of chains (up to 8) and we specified a minimum number of iterations (i.e., 5,000). Default prior settings were used and all trace-plots have been examined to investigate convergence. With Bayesian estimation, missing data is automatically imputed in each step of the Gibbs sampler. So, if 5,000 iterations are requested, missing data is imputed 5,000 times. This makes Bayesian analyses very attractive for dealing with missing data. Since it is beyond the scope of this article to introduce Bayesian statistics, we refer the non-informed reader to, for example, [41], [42], or [43]; for a more technical introduction see [44]. For Bayesian LGM see [45].

When using LGM, the fit of the model should be investigated. Because we used Bayesian statistics default model fit indices, like CFI/TLI/RMSEA, are not available. The Bayesian approach to quantifying model fit is based on posterior predictive *p-values* (*ppp-*value). To compute *ppp*-values, the chi-square value based on the data is compared to the same test statistic, but defined for simulated data. The *ppp*-value is the proportion of chi-square values obtained in the simulated data that exceed that of the actual data. *Ppp*-values around .50 indicate a well-fitting model which can be used to make future predictions. For more information see [46].

The numerical results of a Bayesian analysis might seem identical to the default ML-estimation, but the interpretation is slightly different. As described in Van de Schoot et al. [42]:

“the Bayesian counterpart of the default confidence interval (CI) is the *posterior probability interval* (PPI), also referred to as the *credibility* interval. The PPI is the 95% probability that in the population the parameter lies between the two values. Note, however, that the PPI and the confidence interval may numerically be similar and might serve related inferential goals, but they are not mathematical equivalent and conceptually quite different. We argue that the PPI is easier to communicate because it is actually the probability that a certain parameter lies between two numbers, which is *not* the definition of a classical confidence interval” (p. 8).

Also, Bayesian *p*-values are actually the probability of the null hypothesis being true, which is clearly not the interpretation of a classical *p-*value, but the latter is often misinterpreted as if it were a Bayesian *p*-value (see for example [47]).

## Results

**Figure 1:**
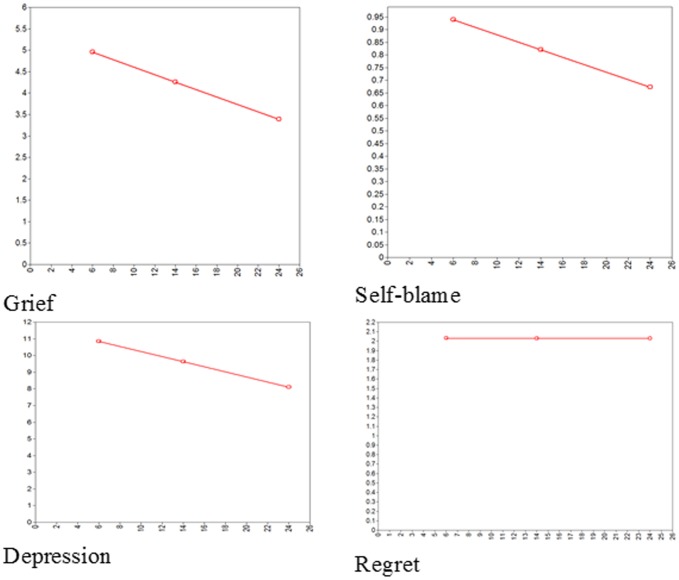
Estimated trajectories of the four variables of interest.

The main results for the four individual LGM analyses of the course of grief, depression, self-blame and regret are displayed in Table 2. See Figure 1 for a graphical representation of the four trajectories. Syntax of the models and data are stored on DANS (Data Archiving and Networked Services; www.dans.knaw.nl). Access to the data can be requested by sending an email to the first author.

**Table 2:** Development of variables over time.

		Intercept					Slope				
	Ppp-value	Means (SD)	P-value*	95% PPI	Variance	95% PPI	Means (SD)	P-value	95% PPI	Variance	95% PPI
Grief	.324	5.476 (0.484)	<.001	4.520–6.410	9.411 (2.845)	5.152–16.142	−0.087 (0.020)	<.001	−0.127– −0.048	0.009 (0.005)	0.001–0.021
Depression	.422	11.764 (1.176)	<.001	9.418–14.074	68.553 (17.485)	42.228–110.802	−0.153 (0.041)	<.001	−0.235− −0.072	0.045 (0.023)	0.010–0.097
Self-blame	.455	1.029 (0.207)	<.001	0.628–1.435	1.700 (0.557)	0.833–3.000	−.015 (.010)	.060	−0.292–0.040	0.002 (0.001)	0.001–0.005
Regret	.458	2.033 (0.294)	<.001	1.453–2.608	3.928 (0.294)	1.453–2.608	.001 (.012)	.494	−0.025–0.024	.005 (0.002)	0.001–0.010

*One-tailed.

All models show a good model fit according to the posterior predictive p-value; all values are close to .50. There is a significant decrease in average grief and depression over the three time points of the study, indicating some adjustment to the loss over the period of the study. In contrast, there is no significant change in average self-blame or average regret over time. All variances around the intercepts and slopes are significant, indicating people show individual differences not only in their starting point (random intercept) but also in their trajectories over time (random slope).

**Figure 2:**
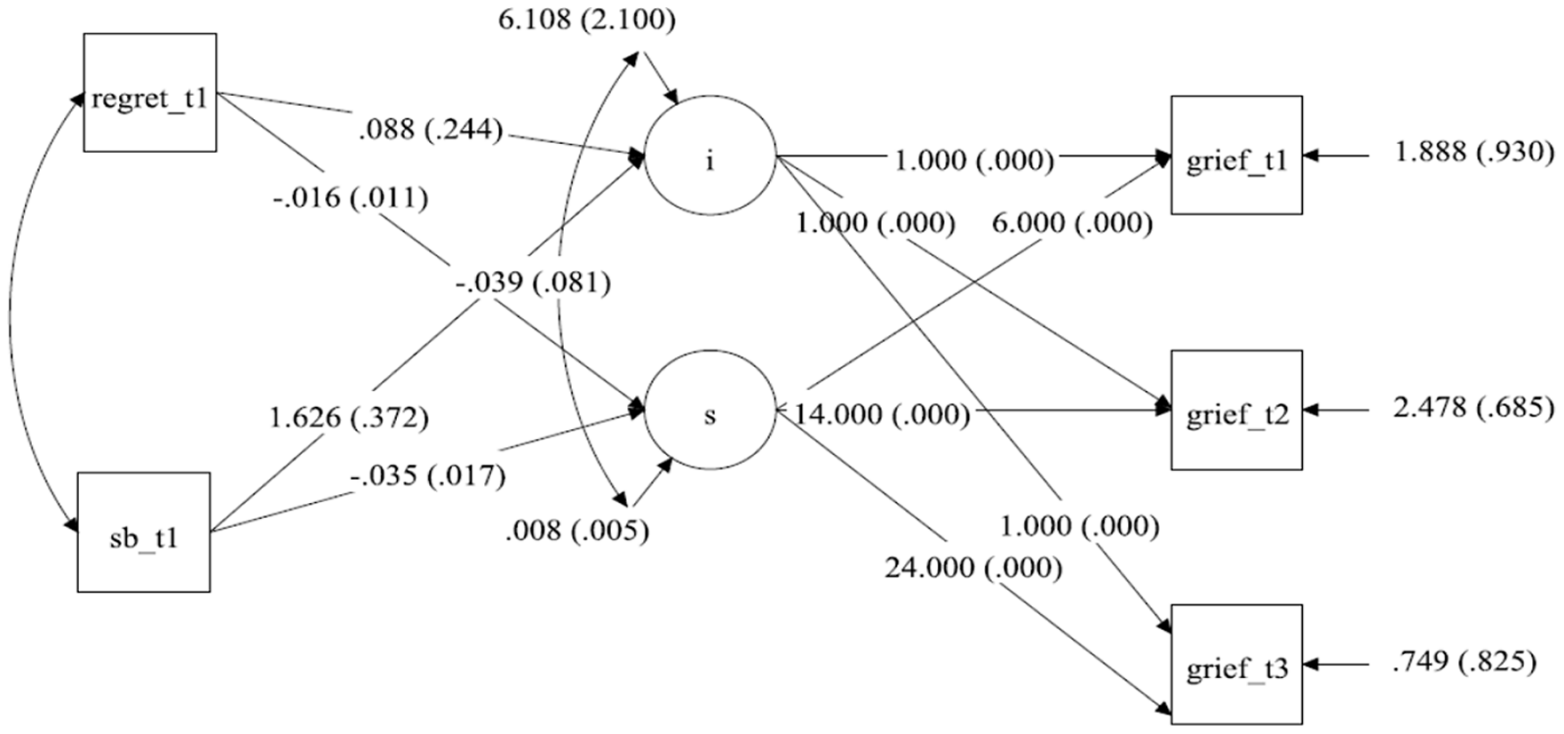
Statistical model with the unstandardized parameters for the development of Grief over time with Self-blame and Regret measured at T1 as predictor for the intercept and slope.

**Figure 3:**
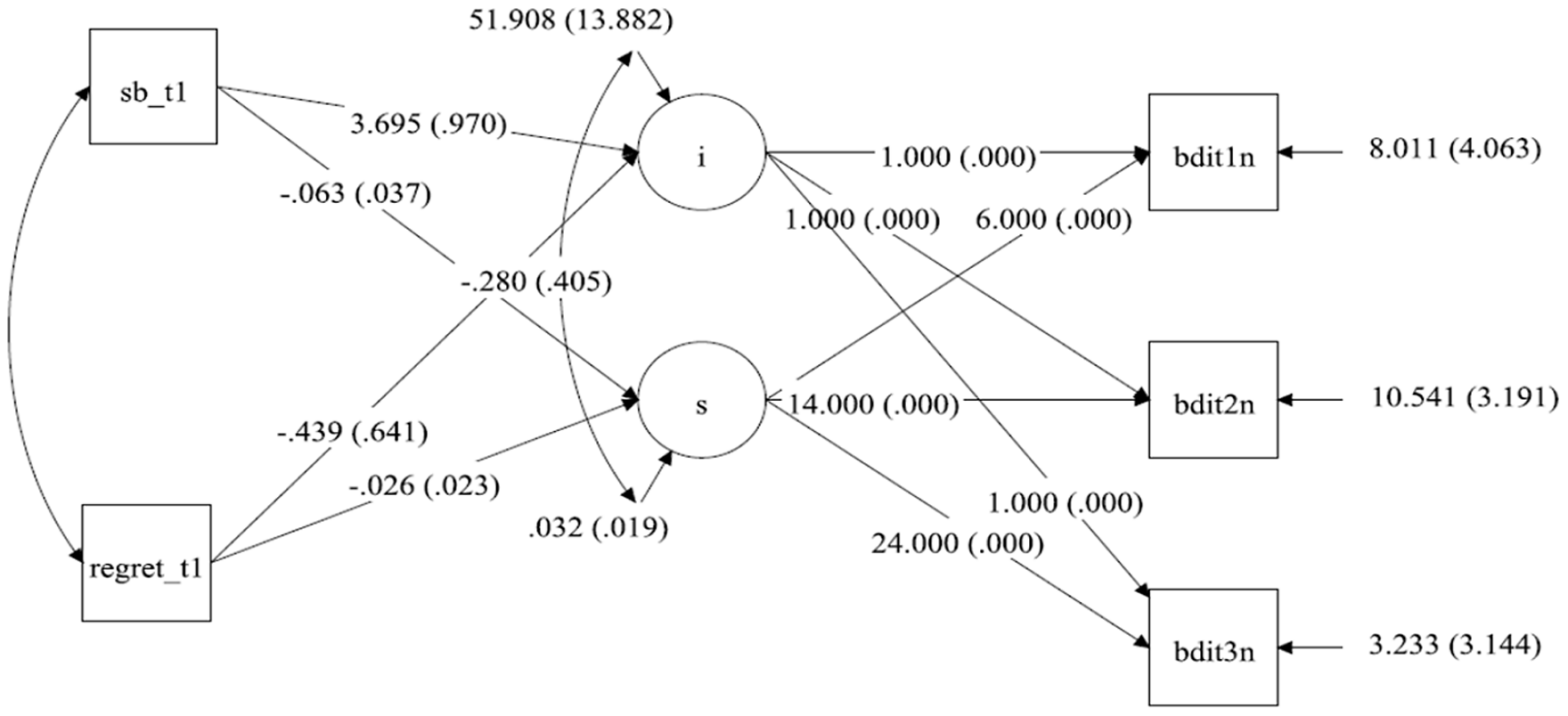
Statistical model with the unstandardized parameters for the development of Depression over time with Self-blame and Regret measured at T1 as predictor for the intercept and slope.

Next, we analysed whether the intercepts and slopes of grief (see Figure 2 *ppp*  = .294) and depression (see Figure 3, *ppp*  = .468) are associated with the two subscales of guilt as measured at T1. We first looked at the association between self-blame at Time 1 (i.e., guilt 1 b; after controlling for Regret) and the intercept of grief (Figure 2) and depression (Figure 3). The higher the value of self-blame at Time 1, the higher are the intercepts of grief (*b* = 1.626, *p*<.001; *R* ^2^ = 42.9% for both predictors) and depression (*b* = 3.695, *p*<.001; *R* ^2^ = 27.0% for both predictors). Thus, the higher the level of self-blame at the beginning of the study, the higher the levels of initial grief and depression.

To assess whether initial self-blame after controlling for regret was related to the course of recovery, we then correlated the initial levels of self-blame to the slopes of grief (Figure 2) and depression (Figure 3). Initial levels of self-blame show a significant negative association with the slope of grief (*b* = −0.035, *p* = .020; *R* ^2^ = 35.2%). The higher the level of self-blame at the beginning of our study, the slower the recovery from grief. Concerning the association between initial self-blame and recovery from depression, although it was on the boundary of being significant (*p* = .049), the PPI includes zero and therefore there is insufficient basis for interpreting this result as significant.

When inspecting the results for Regret as measured at Time 1 (guilt 1 r; after controlling for self-blame), it appeared that in contrast to the pattern observed for self-blame, levels of regret at the beginning of the study were unrelated to both initial levels of grief and depression and the course of recovery from grief and depression (all p-values >.066).

## Discussion

The pattern of the change in grief and depression revealed in Table 2 is consistent with the patterns found in other studies of bereavement, namely that in the course of adjustment to their loss, levels of grief and depression decrease over time [3]. It is interesting to note that the same is not true for self-blame and regret. There is no significant decline in average levels of self-blame or regret over the two-year period (although descriptively, there is some decline in regret that just misses acceptable levels of significance).

More interesting is the evidence for the different roles self-blame and regret appear to play in their association with grief and depression. Because our measures of self-blame and regret share common variance and because we were interested in the univariate association of each variable with grief and depression, we studied the results of self-blame after controlling for regret, and the results for regret after controlling for self-blame. Bereaved individuals with a high level of self-blame at the beginning of our study also experience higher initial levels of grief and show less decline in these symptoms over time (Figure 2). Thus, these individuals form a risk group, having high levels of grief over the loss and experience less decline in their grief symptoms over time. In contrast, recovery from depression is unrelated to initial levels of self-blame (Figure 3). The pattern of findings for regret is most unequivocal: Initial levels of regret are neither predictive of initial levels of grief and depression nor are they related to the recovery process with regard to both outcome variables (Figures 2 & 3).

What conclusions can we draw from these findings? The interesting question to consider here is whether self-blame and regret are mere aspects of the bereavement experience (i.e., correlates) or exert some influence on health outcomes. Although our analyses do not allow one to draw causal conclusions, certain patterns of effects are more consistent with the assumption that a variable plays a causal role than others. One could argue that if self-blame or regret were a (partial) determinant of the impact of the loss experience on health outcomes and adjustment to the loss, then the level of self-blame and regret at the beginning of our study should not only be predictive of the initial level of grief and depression (i.e., intercept) but also of the speed of improvement (i.e., slope).

The strongest positive findings are for the association of self-blame and grief symptoms: High levels of self-blame at the beginning of our study are associated with high initial levels of grief *and* slower decrease of grief symptoms over time. This pattern is consistent with the assumption that self-blame plays a causal role in determining the course of grief symptoms over time. Because there is insufficient evidence that levels of self-blame at Time 1 are associated with the decline in depressive symptoms (even though they are associated with the intercept), it is doubtful that self-blame played a causal role in determining the rate of adjustment with regard to depressive symptoms.

The most unequivocal negative conclusion can be drawn with regard to regret: Initial levels of regret are not only unrelated to the intercepts of grief and depression, they are also unrelated to changes in grief and depression over time. This makes it unlikely that regret plays any causal role in adjustment to loss.

That self-blame appears to predict decline in grief but not depressive symptoms is consistent with the findings of Field, Bonanno, Williams and Horowitz [8], who reported that self-blame was predictive of the rate of decline in grief but not of depressive symptoms. Similarly, Field and Bonanno [7] found that “self-blame was uniquely predictive of grief-specific symptoms across 60 months post loss” (p. 764). But neither the results of the two studies by Field et al. [8], [7], nor our own findings rule out the possibility that the relationship observed between self-blame and grief symptoms is due to some third variable that is related to both self-blame and grief. One plausible third variable would be cause of death. Feeling guilty for not resolving a conflict before the sudden death of one's partner could cause high levels of grief as well as high levels of guilt, without causal relationship between the two. Fortunately, we assessed expectedness of loss in our sample and found it unrelated to self-blame. Furthermore, we found no link between regret and grief/depression, while regret would be likely to be present is such cases too. However, as in all correlational studies, the possibility of third variable explanations cannot be ruled out.

So where does this leave us? The strength of our study is our longitudinal design: the fact that we assessed our variables at three points of time over a period of two years. This design feature allows firmer conclusions than a cross-sectional design. More specifically, it allows us to firmly rule out some hypotheses and to tentatively accept others. Although it is obvious that correlations do not imply causality, people often forget that the opposite is not true: causality does imply correlation. Therefore, the fact that initial levels of regret are not only unrelated to initial symptom levels but are also unrelated to the course of recovery rules out the possibility that regret had a causal influence on grief and depression over the loss of a partner in the present study. Furthermore, even though initial levels of self-blame are associated with levels of depressive symptoms at the start of our study, the fact that they are unrelated to the course of recovery from depression over time is inconsistent with a role as a determinant of depressive symptoms following partner loss. The one pattern that is consistent with a causal role is the finding that initial levels of self-blame are not only predictive of initial levels of grief but also of the course of grief recovery. This finding extends and adds to the evidence (e.g., [7],[8]) that self-blame is a major determinant in shaping the time course of grief over the loss of a partner.

Some further issues need consideration. Could these results be specific to the bereaved cohort under investigation? Although the data were already collected some time ago (cf., [31], [32], [33]), recent developments in statistics enabled analysis of the specific research questions we wanted to address here, using this available data set. We have no reason to assume that the patterns we investigate would be affected by this earlier collection of data. More generally, it is evident that societies have changed in the way that they deal with grief and grieving [48]. Display rules differ: mourning rituals vary across decades and cultures [49]. However, there is also no reason to assume that the essence of the psychological reaction to bereavement is different. To illustrate: a current DSM-5 [50] criterion for Persistent Complex Bereavement Disorder is “Maladaptive appraisals about oneself in relation to the deceased or the death (e.g., self-blame)” (p. 790). Guilt-related phenomena are still considered central.

In similar vein: Can one generalize from our findings, given the relatively low (but typical, as noted above) response rates? Clearly, caution is needed, but again, given that manifestations of guilt in bereavement have been found with consistency across different societies [4], we would hypothesize that similar patterns regarding self-blame and regret would be found elsewhere. This is, then, a topic for further empirical research: the results call for replication in different cultures. Finally, investigation of subgroups and mediators/moderators seems merited. For example, subgroup analyses could examine gender differences; exploration of mediators could include rumination (in the relationship of self-blame and regret to grief and depression).

Some further limitations need to be mentioned. One potential shortcoming of the current study is the limited data set. With conventional estimators, like maximum likelihood estimation, more than 100 participants would have been required to analyse the LGM with enough power. That is, a simulation study was conducted for a multilevel models in the context of comparing countries to find out how many countries are needed to obtain reliable estimates on the country level [37]. With ML-estimation it appeared that only with at least 100 countries was enough precision obtained, see also [51], [52], [53]. With Bayesian estimation, however, Hox et al. showed that reliable results were obtained with only 20–30 countries [54]. These results were recently replicated but for a different multilevel model [55]. Multilevel modeling and latent growth modeling are highly comparable. Besides, Lee and Song argue that with ML estimation one needs at least a ratio of 1∶5 (parameters versus sample size) to obtain reliable results [38]. They furthermore showed that with Bayesian estimation one would only need a ratio of 1∶3 or even 1∶2. Therefore, we conclude that the current limited data set could be analysed with enough estimation precision. However, the reader should bear in mind that as in all observational studies, replication of the current results is warranted.

Furthermore, following the results of our study, further exploration of the causal role of self-blame in the development of grief *complications* is also in order. This seems particularly important in the context of publication of the new manual for the diagnosis of mental disorders, DSM-5 [50], given that it has included “Persistent Complex Bereavement Disorder” as a condition that requires further research before considering it as an established disorder.
